# Comprehensive analysis of mutations associated with rifampicin- and isoniazid-resistant tuberculosis in a high-burden setting

**DOI:** 10.1590/0037-8682-0184-2025

**Published:** 2025-09-22

**Authors:** Rosângela Siqueira de Oliveira, Angela Pires Brandão, Fabiane Maria de Almeida Ferreira, Sonia Maria da Costa, Vera Lucia Maria da Silva, Lucilaine Ferrazoli, Erica Chimara, Juliana Maira Watanabe Pinhata

**Affiliations:** 1 Instituto Adolfo Lutz, São Paulo, SP, Brasil.; 2 Fundação Oswaldo Cruz, Instituto Oswaldo Cruz, Rio de Janeiro, RJ, Brasil.

**Keywords:** Drug susceptibility test, Gene sequencing, GenoType MTBDR*plus*, Multidrug-resistant tuberculosis, Mycobacterium tuberculosis

## Abstract

**Background:**

In this study, we aimed to describe the mutations associated with first-line drug resistance in *Mycobacterium tuberculosis* complex (MTBC) isolates from São Paulo, Brazil, between 2019 and 2021.

**Methods:**

Mutations in the coding regions of *rpoB* and *katG* genes and in the promoter region of the *inhA* gene in MTBC clinical isolates were detected using the GenoType MTBDR*plus* assay (LPA). All mutations inferred by LPA were sequenced.

**Results:**

Of the 13,489 MTBC isolates with valid LPA results, 657 (4.9%) harbored mutations. The overall prevalence rates of rifampicin-resistant (RIF-R) tuberculosis (TB), isoniazid-resistant (INH-R) TB, and multidrug-resistant (MDR) TB were 1.5, 2.0, and 1.2%, respectively. A significant proportion of RIF-R isolates presented inferred *rpoB* mutations (89.1%), most of which were the borderline H445N mutation. The *inhA* promoter C-15T mutation was predominant among the INH-R isolates (52.8%). Most MDR isolates presented *rpoB* S450L + *katG* S315T1 mutations. Gene sequencing identified mutations not included in the catalogue of mutations published by the World Health Organization. Phenotypic drug susceptibility testing on isolates with inferred *rpoB* mutations revealed that the 0.5 µg/mL critical concentration of RIF failed to detect most borderline mutations when using the BACTEC MGIT 960 system.

**Conclusions:**

These findings emphasize the need for continuous surveillance and the integration of molecular and phenotypic methods to ensure an accurate detection and management of drug-resistant TB in high-burden settings.

## INTRODUCTION

Despite being a preventable and curable disease, in 2023, tuberculosis (TB) regained its position as the leading cause of death worldwide from a single infectious agent, after being temporarily surpassed by COVID-19^1^. In 2023, 175,923 individuals were diagnosed and treated for multidrug-resistant (MDR) or rifampicin-resistant (RIF-R) TB, representing only 44% of the estimated 400,000 cases of MDR/RIF-R-TB globally^1^.

Brazil is ranked among the 30 high-burden countries, which together accounted for 87% of the global cases of TB in 2023^1^, wherein Brazil reported over 80,000 new TB cases.In 2022, the country recorded approximately 5,800 TB-related deaths^2^. The state of São Paulo contributes significantly to this burden, accounting for approximately 25% of the national burden, with 19,571 new TB cases in 2023^2^.

Line probe assays (LPAs) are molecular tests performed directly on pulmonary samples or clinical isolates. The GenoType MTBDR*plus* assay (Bruker-Hain Lifescience GmbH, Nehren, Germany) helps detect *Mycobacterium tuberculosis* complex (MTBC) and associated resistance to isoniazid (INH) and RIF, i.e., MDR-TB. 

There is limited information on the molecular mutations underlying drug resistance in TB in Brazil, with most published studies describing local distribution patterns^3^ ^-^ ^9^. In this study, we aimed to describe the mutations associated with RIF-R and INH-R in MTBC isolates and assess the prevalence of first-line drug-resistant TB in São Paulo.

## METHODS

### Ethics statement

This study was approved by the Technical Scientific Council (approval number: 45M/2020) and Research Ethics Committee (approval number: 6.110.210) of our institution.

### Clinical isolates

The network of TB laboratories in São Paulo performs the Xpert MTB/RIF Ultra assay (Cepheid, Sunnyvale, CA, USA), culture, and smear microscopy when indicated. Mycobacterial cultures are then sent to our laboratory for species identification and drug susceptibility testing (DST). Most laboratories provide Xpert results on the requisition form accompanying the submitted cultures. In our laboratory, isolates are initially assessed based on their growth characteristics to presumptively determine whether they belong to MTBC or are non-tuberculous mycobacteria^10^ ^,^ ^11^. All presumptive MTBC isolates from patients meeting the DST criteria established in Brazil^12^, routinely tested using LPA between January 2019 and December 2021, were included in this study. Additional isolates from the same patient were included only when their resistance results differed.

### GenoType MTBDR*plus* v. 2.0

DNA extraction and PCR were performed according to the instructions specified in the GenoType MTBDR*plus* assay kit manual^13^. The PCR products were hybridized with probes immobilized on strips using the GT-Blot 48 automated system (Bruker-Hain Lifescience GmbH, Germany). Results were interpreted using the GenoScan software (Bruker-Hain Lifescience) and released by a professional.

An inconclusive result was defined when the TUB (probe that identifies the MTBC) band was positive, but the wild-type (WT) bands were faint, and no mutation (MUT) bands developed owing to low bacterial load, which hindered drug resistance detection. When the results remained inconclusive after repeated testing, a new culture was requested.

A mutation was inferred in the absence of hybridization with both the WT and corresponding MUT probe(s). As LPA targets the 81-bp RIF-R-determining region (RRDR) of *rpoB*, isolates with inferred *rpoB* mutations were classified as RIF-R^14^. Similarly, isolates with mutations in *katG* and the *inhA* promoter were classified as INH-R, even when the mutation was not listed in the catalogue of mutations published by the World Health Organization (WHO)^15^.

An unexpected result occurred when two *rpoB* WT probes were expected to be positive, but only one was (e.g., WT3 positive, WT4 absent), which is not anticipated by the manufacturer. These cases were considered RIF-R when the clinical specimen was confirmed as RIF-R in the Xpert Ultra assay (Cepheid, USA). Otherwise, a cautionary note suggesting a silent mutation was included in the report. If the Xpert test result was unavailable, a phenotypic DST (pDST) was performed. Doubtful results were obtained when both the WT and corresponding MUT band(s) were faintly positive.

### Gene sequencing

Sanger sequencing was performed to characterize the inferred mutations in the LPA gene targets and confirm doubtful results. For *rpoB*, the pair of primers amplified a 350-bp fragment covering the RRDR (positions 1,184-1,533 from the start codon). *katG* was sequenced using five pairs of primers from position −135 upstream of the *katG* start codon to +431 nucleotides downstream from the gene ending. The primers for the *inhA* promoter amplified a 248-bp fragment spanning positions −168 to +80 from the start codon of *fabG1* ^7^.

The reaction mixes (25 µL) contained 1× buffer, 1.75 mM MgCl_2_ for *rpoB* (1.5 mM for *katG*/*inhA*), 0.75 U Taq polymerase (GoTaq G2 Flexi; Promega Corporation, Madison, WI, USA), 200 µM of each dNTP (dNTP mix; Promega, USA), 10 pmol primers for *rpoB* (5 pmol for katG/*inhA*), approximately 50 ng of DNA template, and nuclease-free water (Qiagen GmbH, Hilden, Germany). Amplification included initial denaturation at 94°C for 5 min, followed by 40 cycles of denaturation at 94°C for 1 min, annealing at 60°C for *rpoB* (62°C for *katG*; 64°C for *inhA*) for 1 min, and extension at 72°C for 1 min, with a final extension at 72°C for 10 min. Primers and unincorporated nucleotides were enzymatically removed (ExoSAP-it; Affymetrix, Inc., Santa Clara, CA, USA), and the amplicons were sequenced using the BigDye Terminator v3.1 (Applied Biosystems, Foster City, CA, USA). The sequence data generated on an ABI 3130×L Genetic Analyzer (Applied Biosystems, USA) were analyzed using BioEdit v7.2.5, MUBII-TB-DB, and BLAST tools^7^. The DNA sequences of all sequenced isolates were deposited in GenBank (https://www.ncbi.nlm.nih.gov) under the identification PRJNA888434.

### pDST using BACTEC MGIT 960

pDST was performed to complement the LPA and Xpert results, when available, for interpreting RIF resistance. MTBC cultures were routinely tested for drug susceptibility using the BD BACTEC MGIT 960 IR Kit (Becton, Dickinson and Company, Sparks, MD, USA)^16^. INH was tested at the critical concentration of 0.1 µg/mL. From January 2019 to May 2021, RIF was tested at the critical concentration of 1.0 µg/mL; however, the critical concentration was later adjusted to 0.5 µg/mL for the June-December 2021 period, following WHO recommendations^17^.

In this study, we present only the pDST results for the isolates tested at the WHO-recommended RIF critical concentration of 0.5 µg/mL. Based on the WHO recommendations to report any *rpoB* mutation detected by LPA as RIF-R, not all isolates with inferred mutations in this study were subjected to pDST^18^.

### Declaration of the use of generative AI and AI-assisted technologies in the writing process

During the preparation of this manuscript, the authors used ChatGPT (OpenAI) to assist in improving the clarity and readability of the text. All outputs from the tool were carefully reviewed, revised, and approved by the authors, who assume full responsibility for the content of the final published article.

## RESULTS

Between January 2019 and December 2021, our laboratory received 23,696 cultured isolates: 8,710 (36.7%) in 2019, 7,526 (31.8%) in 2020, and 7,460 (31.5%) in 2021. Among these, 15,724 (66.4%) isolates were analyzed using LPA. Of these, 5,855 (37.2%) were received in 2019, 4,857 (30.9%) in 2020, and 5,012 (31.9%) in 2021. Among the tested isolates, 13,489 (85.8%) were identified as MTBC with valid resistance results, 411 (2.6%) were non-MTBC, 20 (0.1%) were inconclusive, and 1,804 (11.5%) were additional isolates from patients already included in the study. Of the 13,489 MTBC isolates, 657 (4.9%) harbored mutations, with 254 (38.7%) exhibiting 258 inferred mutations in any of the three genes. Fourteen of the 254 isolates were not sequenced because their DNA samples were unavailable, resulting in 240 sequenced isolates. The LPA and sequencing results for all inferred mutations are shown in Table 1. 

**TABLE 1:** Mutations identified by DNA sequencing of the *rpoB* and *katG* coding regions and the *inh*A promoter region in 240 of 254 *Mycobacterium tuberculosis* complex isolates presenting 258 inferred mutations detected using the Genotype MTBDR*plus* assay.

**Genotype MTBDR*plus* Genes and mutation probes**	Total N/No.	%	Mutations identified by gene sequencing*	Total N/No.	%
***rpoB* gene**	**230/258**	**89.1**		**216/244**	***88.5***
WT1 neg	3/230	1.3	T427T (1281 c>t) - silent	3/216	*1.4*
WT2 neg	22/230	9.6	L430P (1289 t>c)^c^	21/216	*9.7*
			L430P (1289 t>c)^c^ + F433dup (1297_1298ins ttc)^a^	1/216	*0.5*
WT2,3 neg	4/230	1.7	L430Lfs*10 (1290_1299del gagccaattc)^e^	1/216	*0.5*
			Q432K (1294 c>a)^a^	1/216	*0.5*
			Q432P (1295 a>c)^a^	1/216	*0.5*
			Q432R (1295 a>g)^e^	1/216	*0.5*
WT2,3,4 neg	1/230	0.4	Q432Q (1296 a>g, silent) + F433_D435del (1297_1305del ttcatggac)^b^	1/216	*0.5*
WT3,4 neg, WT7+MUT2A pos (H445Y HR)	2/230	0.9	Q432H (1296 a>c) + F433_D435del (1297_1305del ttcatggac)^e^	2/216	*0.9*
WT3 neg	30/230	13.0	F433F (1299 c>t) - silent	23/216	*10.6*
			F433dup (1297_1298ins ttc)^a^	1/216	*0.5*
			F433dup (1297_1298ins ttc)^a^ + **P454S** (1360 c>t)^d^	2/216	*0.9*
			N/D	(4)	*-*
WT3,4 neg	4/230	1.7	M434Qfs (1300_1305del atggac)^e^	1/216	*0.5*
			D435Y (1303 g>t)^c^	3/216	*1.4*
WT3,4,7 neg	2/230	0.9	M434I (1302 g>a)^b^ + H445N (1333 c>a)^c^	2/216	*0.9*
WT5 neg^‡^	9/230	3.9	P439L (1316 c>t)^b^	7/216	*3.2*
			N/D	(2)	*-*
WT6 neg	1/230	0.4	S441S (1323 g>a) - silent	1/216	*0.5*
WT6 neg, WT3,4+MUT1 weak (D435Y HR)	1/230	0.4	L443S (1328 t>c)^b^	1/216	*0.5*
WT7 neg^§^	130/230	56.5	T444T (1332 c>g, silent) + H445S (1333 ca>tc)^c^ + K446Q (1336ª>c)^b^	1/216	*0.5*
			H445C (1333 ca>tg)^a^	2/216	*0.9*
			H445L (1334 a>t)^c^	3/216	*1.4*
			H445N (1333 c>a)^c^	115/216	*53.2*
			H445R (1334 a>g)^a^	1/216	*0.5*
			H445S (1333 ca>ag)^c^	1/216	*0.5*
			N/D	(7)	*-*
WT8 weak, MUT2B pos (H445D)^a^	1/230	0.4	H445D (1333 c>g)^a^	1/216	*0.5*
WT8 neg^†^	20/230	8.7	S450L (1349 c>t)^a^	5/216	*2.3*
			S450W (1349 c>g)^a^	6/216	*2.8*
			**Q409R** (1226 a>g)^d^ + S450W (1349 c>g)^a^	1/216	*0.5*
			L452P (1355 t>c)^a^	7/216	*3.2*
			N/D	(1)	*-*
***katG* gene**	**17/258**	6.6		**17/244**	***7.0***
WT neg	14/17	82.4	S315G (943 a>g)^d^	3/17	*17.6*
			S315I (944 g>t)^b^ + A379V (1136 c>t)^d^	1/17	*5.9*
			S315N (944 g>a)^a^	4/17	*23.5*
			S315N (944 g>a)^a^ + A379T (1135 g>a)^d^	5/17	*29.4*
			S315T (944 gc>cg)^a^	1/17	*5.9*
Loccus, WT, MUT1, MUT2 neg	3/17	17.6	*katG* del (partial matching with reference)^e^	3/17	*17.6*
***inhA* promoter region**	**11/258**	4.3		**11/244**	***4.5***
WT1 neg	4/11	36.7	g-17t^b^	2/11	*18.2*
			g-19t^d^	1/11	*9.1*
			c-20g^e^	1/11	*9.1*
WT2 neg	7/11	63.3	t-8g^b^	6/11	*54.5*
			WT	1/11	*9.1*

**WT:** wild type; **neg:** negative, **i.e:** lack of binding to a probe; **pos:** positive signal; **HR:** heteroresistance; **N/D:** not done; underlined are mutations for which MTBDR*plus* has specific probes; in bold are mutations outside the rifampicin-resistance determining region; ^‡^ an isolate P439L had additional weak signals at wt7-mut2a bands; ^§^ three H445N HR results, an isolate H445N had additional wt3,4 weak and another one, wt4,8 weak bands; ^†^ one S450L sample had additional wt3,4-mut1 and wt7-mut2a weak bands; one S450W was HR. * Mutation classification based on the catalogue of mutations published by the World Health Organization (2023): ^a^associated with resistance, ^b^associated with resistance - interim, ^c^associated with resistance (borderline), ^d^uncertain significance, ^e^not on catalogue.

Most inferred mutations were detected in *rpoB* (89.1%, 230/258). In the LPA, the most frequently negative *rpoB* probes were WT7 (56.5%; 130/230), WT3 (13.0%; 30/230), and WT2 (9.6%; 22/230) (Table 1). Of the 244 sequenced targets, 216 (88.5%) were *rpoB*. The most common *rpoB* mutation was H445N (missing the WT7 band), accounting for 53.2% (115/216) of the inferred *rpoB* mutations by LPA and 47.1% (115/244) of all these mutations. Four mutations were not listed in the WHO catalogue, including indels (L430Lfs*10, Q432Hfs, and M434Qfs) and point mutations (Q432R). In addition to F443F (WT3-negative), the S441S silent mutation was an unexpected LPA result (WT6-negative). Conversely, the silent mutation T427T was expected (WT1-negative).

Six mutation types were identified in 6.6% isolates presenting inferred *katG* mutations (17/258). Most mutations (14/17, 82.4%) occurred at codon 315. Mutations in three (17.6%) isolates were likely due to large genomic deletions, as their *katG* sequences only partially matched the reference genome: in the first isolate, a fragment covering nucleotides 408-2,075 was identified; the second isolate yielded a sequenced fragment covering nucleotides 1,404-1,707; whereas the third isolate yielded no fragment of the *katG* gene (Table 1). 

In LPA, a WT2-negative probe (7/11; 63.3%) was the most common among the inferred mutations in the *inhA* promoter, accounting for 4.3% (11/258) of these mutations. Among the four changes identified, C-20G (WT1-negative) was not listed in the WHO catalogue (Table 1). 

The mutation patterns detected in the *rpoB* and*katG* genes and the *inhA* promoter in the 657 isolates are presented in Table 2. According to the LPA, 234 (35.6%) isolates were classified as RIF-R, with the majority (195/234; 83.3%) exhibiting inferred mutations. Of the 14 isolates that were not sequenced (13 RIF-R and 1 MDR), 7,4, and 2 isolates exhibited WT7-, WT3-, and WT5-negative results, respectively, and one isolate exhibited WT8-negative results. Among the 222 RIF-R isolates with identified mutations, the most common were H445N (51.4%), F433F, and S450L (10.4% each) (Table 2). Twenty-seven (12.2%) isolates harbored single silent mutations.

**TABLE 2:** Pattern of mutations in the *rpoB* and *katG* coding regions and the *inhA* promoter regions in *Mycobacterium tuberculosis* complex isolates, São Paulo, Brazil, between 2019 and 2021.

Resistance detected using LPA	Mutated gene	*N*	***N* HR**	Mutation profile of the isolates			Discordances in resistance after
				rpoB*	katG*	*inhA* promoter*	sequencing
INH-R (*n* = 265)	*katG* (*n* = 116)	1	-	WT	S315I^b^ + A379V^d^	WT	
		3	-	WT	S315G^d^	WT	
		1	-	WT	S315N^a^	WT	
		106	6	WT	S315T1 ^a^	WT	
		2	-	WT	S315T2 ^a^	WT	
		1	-	WT	S315T3^a^	WT	
		2	-	WT	*katG* del^e^	WT	
	*inhA* promoter (*n* = 148)	1	-	WT	WT	t-8c ^a^	
		5	-	WT	WT	t-8g^b^	
		139	13	WT	WT	c-15t ^a^	False-WT by sequencing (*n* = 1, HR)
		1	1	WT	WT	a-16g ^a^	
		1	-	WT	WT	g-19t^d^	
		1	-	WT	WT	c-20g	
	*katG* + *inhA* promoter (*n* = 1)	1	-	WT	S315T1 ^a^	c-15t ^a^	
RIF-R (*n* = 234)		1	-	**Q409R** ^d^ + S450W^a^	WT	WT	-
		3	-	T427T	WT	WT	False-RIF-R by LPA (n=3)
		1	-	L430Lfs*10^e^	WT	WT	-
		19	-	L430P^c^	WT	WT	-
		1	-	L430P^c^ + F433dup^a^	WT	WT	-
		1	-	Q432H fs^e^	WT	WT	-
		1	-	Q432K^a^	WT	WT	-
		1	-	Q432Q + F433_D435del^b^	WT	WT	-
		1	-	Q432R^e^	WT	WT	-
		23	-	F433F	WT	WT	False-RIF-R by LPA (n=23)
		1	-	F433dup^a^	WT	WT	-
		1	-	M434Q fs^e^	WT	WT	-
		2	1	D435V	WT	WT	-
		2	-	D435Y^c^	WT	WT	-
		7	-	P439L^b^	WT	WT	-
		1	-	S441S	WT	WT	False-RIF-R by LPA (n=1)
		8	1	H445D ^a^	WT	WT	-
		1	1	H445D ^a^ + H445Y	WT	WT	-
		114	3	H445N^c^	WT	WT	-
		5	-	H445Y	WT	WT	-
		23	4	S450L ^a^	WT	WT	-
		1	-	S450W^a^	WT	WT	-
		3	-	L452P^a^	WT	WT	-
		4	-	WT3 neg	WT	WT	N/S
		2	-	WT5 neg	WT	WT	N/S
		7		WT7 neg	WT	WT	N/S
MDR (*n* = 158)	*rpoB* + *kat*G (*n* = 115)	1	-	Q432H fs^e^	S315T1 ^a^	WT	-
		1	-	Q432P^a^	S315T1 ^a^	WT	-
		2	-	F433dup^a^ + **P454S** ^d^	S315T1 ^a^	WT	-
		5	-	D435V	S315T1 ^a^	WT	-
		1	-	D435Y	S315T1 ^a^	WT	-
		1	-	K443S^b^	S315T1 ^a^	WT	-
		1	-	T444T + H445S^c^ + K446Q^b^	S315T1 ^a^	WT	-
		2	-	H445C^a^	S315T1 ^a^	WT	-
		4	-	H445D ^a^	S315T1 ^a^	WT	-
		2	-	H445D ^a^ + H445Y	S315T1 ^a^	WT	-
		2	-	H445L^c^	S315T1 ^a^	WT	-
		1	-	H445R^a^	S315T1 ^a^	WT	-
		1	-	H445S^c^	S315T1 ^a^	WT	-
		5	2	H445Y	S315T1 ^a^	WT	-
		3	-	S450L ^a^	S315N^a^	WT	-
		74	2	S450L ^a^	S315T1 ^a^	WT	-
		1	-	S450L ^a^	*katG* del^e^	WT	-
		4	-	S450W^a^	S315T1 ^a^	WT	-
		3	-	L452P^a^	S315T1 ^a^	WT	-
		1	-	WT8 neg	S315T1 ^a^	WT	N/S
	*rpoB + inhA* promoter (*n* = 30)	2	-	L430P^c^	WT	c-15t^a^	-
		2	1	H445Y	WT	c-15t^a^	-
		1	-	H445D^a^	WT	WT2 neg	False-WT by sequencing (*n* = 1, HR)
		1	1	H445Y + S450L ^a^	WT	c-15t ^a^	-
		22	3	S450L ^a^	WT	c-15t ^a^	-
		1	1	S450W^a^	WT	t-8g^b^	-
		1	-	L452P^a^	WT	c-15t ^a^	-
	*rpoB* + *katG* + *inhA* promoter (*n* = 13)	1	-	H445D ^a^	S315T1 ^a^	g-17t^b^	-
		2	-	H445Y	S315T1 ^a^	c-15t ^a^	-
		1	-	H445L^c^	S315T1 ^a^	g-17t^b^	-
		1	-	H445N^c^	S315T1 ^a^	c-15t ^a^	-
		2	-	M434I^b^ + H445N^c^	S315T1 ^a^	c-15t ^a^	-
		5	-	S450L ^a^	S315N^a^ + A379T^d^	c-15t ^a^	-
		1	-	S450L ^a^	S315T1 ^a^	c-15t ^a^	-
No resistance	-	12832	-	WT	WT	WT	-

**LPA:** Genotype MTBDR*plus* line probe assay; **INH:** isoniazid; **RIF:** rifampicin; **HR:** heteroresistance; **N/S:** not sequenced; **HR:** heteroresistance; **WT:** wild type; **neg:** negative (lack of binding to a probe); underlined are mutations for which MTBDR*plus* has specific probes; in bold are mutations outside the rifampicin-resistance determining region. * Mutation classification based on the catalogue of mutations published by the World Health Organization (2023): ^a^associated with resistance, ^b^associated with resistance - interim, ^c^associated with resistance (borderline), ^d^uncertain significance, ^e^not on catalogue.

Regarding the mutations in 265 (40.3%) INH-R isolates, 148 (55.8%) and 116 (43.8%) isolates exhibited mutations in the *inhA* promoter and *katG*, respectively; however, one (0.4%) isolate exhibited mutations in both genes. The most common were the *inhA* promoter C-15T (52.8%) and *katG* S315T (g>c) (40.4%) substitutions (Table 2).

Among the 158 (24.1%) MDR isolates detected by LPA, most mutations (115/158; 72.8%) occurred in *rpoB* and *katG*, with the most common *rpoB*_S450L/*katG_*S315T being identified in 74 (46.8%) MDR isolates. Inferred *katG* mutations were detected in four isolates with the *rpoB* S450L mutation, whereas inferred mutations in *rpoB* were identified in 22 isolates with the *katG* S315T mutation. Among the 30 (19.0%) MDR isolates with mutations in both *rpoB* and *inhA* promoter detected by LPA, the most common was *rpoB*_S450L/*inhA*_C-15T, identified in 22 (13.9%) isolates, including three heteroresistant *rpoB* cases (S450L+WT). Four inferred mutations were detected in *rpoB* and one in the *inhA* promoter. Additionally, 13 MDR isolates (8.2%) exhibited mutations in all three target genes. The most frequent profile in this group was *rpoB*_S450L/*katG_*S315N+A379T/*inhA*_C-15T, observed in five isolates (Table 2). 

Certain mutations exhibited markedly different distributions between RIF-R and MDR isolates. Notably, the H445N mutation was detected in 48.5% (114/235) of RIF-R isolates but only 1.9% (3/158) of MDR isolates (*p*<.0001), whereas the S450L mutation occurred in 67.7% (107/158) of MDR isolates and 9.8% (23/235) of RIF-R isolates (*p*<.0001). Silent mutations occurring as single mutations were observed exclusively in RIF-R isolates (Table 2).

As shown in Table 2, the proportion of heteroresistance detected by LPA was low in our study, being found in 10/234 (4.3%) RIF-R, 20/265 (7.5%) INH-R, and 10/158 (6.3%) MDR isolates. The sequencing of two doubtful results revealed a mix of WT and S315T mutation in an isolate with *katG* WT-MUT weak positive bands in LPA, but missed the C-15T mutation in a heteroresistant culture with WT1-MUT1 weak positive bands in LPA.

Assuming that all mutations found in this study were associated with RIF-R or INH-R, excluding silent mutations, the drug resistance prevalence in the 3-year study period was 1.5% (207/13,489) for RIF-R-TB, 2.0% (265/13,489) for INH-R-TB, and 1.2% (157/13,489) for MDR-TB. RIF-R-TB prevalence remained steady at 1.5% (76/5,174) in 2019, 1.5% (61/4,060) in 2020, and 1.6% (70/4,255) in 2021. INH-R-TB showed slight fluctuations and was 2.0% (105/5,174) in 2019, 1.7% (68/4,060) in 2020, and 2.2% (92/4,225) in 2021. MDR-TB prevalence remained stable at 1.2% (62/5,174) in 2019, 1.1% (44/4,060) in 2020, and 1.2% (52/4,255) in 2021.

Overall, 123 among the 230 isolates with inferred *rpoB* mutations (53.5%) underwent RIF testing using MGIT, of which 111 showed valid results: 76 (68.5%) and 35 (31.5%) at 1.0 and 0.5 µg/mL, respectively. Only the isolates tested at RIF 0.5 µg/mL are shown in Table 3, which describes pDST results for 39 isolates with inferred mutations in LPA (35 in *rpoB,* 1 in *katG,* and 3 in *inhA*). Among the 35 isolates presenting inferred *rpoB* mutations, 21 were borderline mutations (19 H445N and 2 L430P), and all tested RIF susceptible on MGIT. Of the two isolates with *rpoB* mutations associated with interim resistance the isolate with the Q432R mutation tested MDR on MGIT (notably, this isolate was INH susceptible on LPA), and the isolate with the P439L mutation was RIF susceptible on MGIT. The two isolates with mutations associated with RIF-R according to the WHO catalogue (*rpoB* Q432K - this isolate did not show mutations in either the *katG* coding region or the *inhA* promoter; and H445L - this isolate also had the *inhA* promoter G-17T mutation) were phenotypically MDR. 

**TABLE 3:** Results for the phenotypic drug susceptibility testing (DST) using the BACTEC MGIT 960 system of *Mycobacterium tuberculosis* clinical isolates with mutations inferred in the GenoType MTBDR*plus* line probe assay (LPA)*.*

**LPA results (*rpoB/katG/inhA* profile)**	Gene sequencing results	WHO mutations catalogue (2023)	N	Phenotypic DST results
RIF R, INH S (WT2 neg/WT/WT)	*rpoB* L430P	Assoc. w R (borderline)	2	RIF S, INH S
RIF R, INH S (WT2,3 neg/WT/WT)	*rpoB* Q432K	Assoc. w R	1	MDR
	*rpoB* Q432R	Assoc. w R - interim	1	MDR
RIF R, INH S (WT3 neg/WT/WT)	*rpoB* F433F (silent)	Not assoc. w R	8	RIF S, INH S
RIF R, INH S (WT5 neg/WT/WT)	*rpoB* P439L	Assoc. w R - interim	1	RIF S, INH S
RIF R, INH S (WT7 neg/WT/WT)	*rpoB* H445N	Assoc. w R (borderline)	18	RIF S, INH S
MDR (WT6 neg, WT3,4+MUT1 faint /S315T1/WT)	*rpoB* L443S	Assoc. w R - interim	1	RIF S, INH R
MDR (WT7 neg/S315T1/WT)	*rpoB* H445R	Assoc. w R	1	MDR
MDR (WT7 neg/S315T1/WT1 neg)	*rpoB* H445L, *inhA* G-17T	Assoc. w R (*rpoB*), uncertain significance (*inhA*)	1	MDR
MDR (WT8 neg/S315T1/WT)	*rpoB* S450W	Assoc. w R	1	MDR
INH R (WT/Locus, WT neg/WT)	*katG* does not align to reference	Not on catalogue*	1	INH R
INH R (WT/WT/WT1 neg)	*inhA* G-19T	Uncertain significance	1	RIF S, INH S
	*inhA* C-20G	Not on catalogue	1	INH R
INH R (WT/WT/WT2 neg)	*inhA* T-8G	Assoc. w R - interim	1	RIF S, INH S
**Total**	**-**	**-**	**39**	**-**

**WHO:** World Health Organization; **INH:** isoniazid; **RIF:** rifampicin; **neg:** negative (absence of the respective band); **WT:** wild type; **S:** susceptible; **R:** resistant; **Assoc. w R:** mutation associated with resistance; **Not assoc. w R:** mutation not associated with resistance; underlined are inferred mutations; *probably a loss-of-function mutation due to gene deletion

**FIGURE 1:**
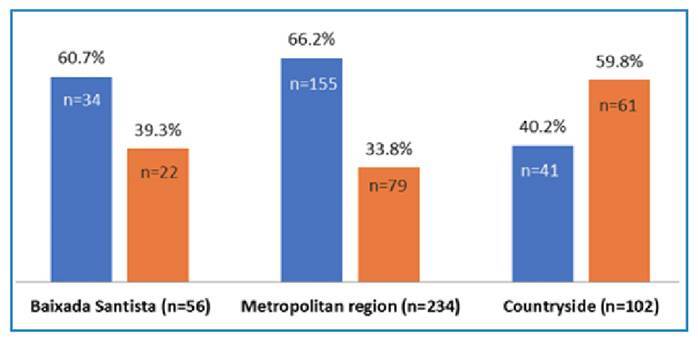
Distribution of canonical (orange) and inferred (blue) *rpoB* mutations in *Mycobacterium tuberculosis* isolates across the state of São Paulo, Brazil. In Baixada Santista (n=56) and the Metropolitan region (n=234), inferred mutations were more frequent, representing 60.7% (n=34) and 66.2% (n=155) of cases, respectively, while canonical mutations accounted for 39.3% (n=22) and 33.8% (n=79). In contrast, in the Countryside (n=102), canonical mutations predominated (59.8%, n=61) compared to inferred mutations (40.2%, n=41). These data reveal geographic heterogeneity in mutation profiles, with inferred mutations more common in urbanized, high-incidence areas and canonical mutations more prevalent in less densely populated regions.

One isolate with an inferred *katG* mutation (locus and WT bands absent) was INH-R, as confirmed by pDST, and harbored a *katG* deletion, likely a loss-of-function mutation associated with resistance. Three isolates exhibited inferred mutations in the *inhA* promoter (C-20G, G-19T, and T-8G), and only the isolate harboring the C-20G mutation (not in the catalogue) tested INH-R using MGIT (Table 3). The analysis of *rpoB* mutations in 392 isolates (230 with inferred and 162 with canonical mutations) across the state of São Paulo revealed that inferred mutations were more frequent in Baixada Santista (60.7%) and the São Paulo Metropolitan Region (66.2%), whereas canonical mutations predominated in the countryside (Figure 1). 

## DISCUSSION

This study provides a comprehensive molecular characterization of RIF-R, INH-R, and MDR MTBC clinical isolates from São Paulo over a 3-year period, overlapping the COVID-19 pandemic. In total, 13,635 isolates were analyzed using LPA, revealing valuable insights into the prevalence of drug resistance. In 2019, the number of mycobacterial isolates analyzed was higher than in 2020 and 2021 owing to the impact of the COVID-19 pandemic on TB diagnosis. Pontes et al. (2024) reported a significant decline in TB notifications in São Paulo, highlighting the disruptive effects of the pandemic on healthcare services^19^.

The overall prevalences of RIF-R-TB, INH-R-TB, and MDR-TB were 1.5, 2.0, and 1.2%, respectively. Although RIF-R and MDR prevalences remained stable, that of INH-R-TB fluctuated slightly, with a decrease in 2020, followed by an increase in 2021. Brazil is considered a high-burden country for TB and TB/HIV co-infection; however, drug resistance rates remain relatively low, a trend also reflected in the resistance rates observed in São Paulo.

Mutational analysis revealed that a significant proportion (89.1%) of RIF-R isolates (excluding 27 isolates with silent mutations) harbored inferred mutations, most of them identified as the borderline H445N. The L430P borderline mutation was also frequent. The high occurrence of borderline *rpoB* mutations observed in our setting has been noted since the implementation of Xpert MTB/RIF in 2014, when these mutations were linked to discrepancies between molecular and pDST results^4^ ^,^ ^7^. The frequency of the S450L mutation, identified in 9.6% of RIF-R cases in this study, aligns with previous findings^7^ ^,^ ^20^.

Discordant results for RIF, characterized by genotypically resistant but phenotypically susceptible *M. tuberculosis* strains, have frequently been reported and present significant challenges for treatment and clinical management. These discordances are often caused by borderline mutations in *rpoB*. Studies have demonstrated that such mutations can be clinically relevant and may significantly affect treatment outcomes in patients with RIF-R or MDR-TB^21^ ^,^ ^22^. Therefore, detecting borderline mutations is as critical as identifying the mutations associated with high-level resistance.

A previous study by our group reported that the prevalence of MTBC isolates with genotypic resistance to RIF but discordant phenotypic susceptibility was 55.1% in São Paulo between 2014 and 2017. Among them, 75.5% of the sequenced and restriction fragment length polymorphism-genotyped isolates were grouped into molecular clusters, mostly associated with *rpoB* mutations. The most prevalent mutation was the borderline H445N, which was frequently found in isolates circulating in Baixada Santista and the São Paulo Metropolitan Region^4^. Although molecular typing of the isolates was not performed in the present study, the inferred mutations were observed more frequently in these two regions.

A study conducted in Namibia using whole-genome sequencing (WGS) identified the L430P borderline mutation in clustered isolates, suggesting a high transmission potential for these strains^23^. Lempens et al.^21^, also using WGS, concluded that isolates harboring borderline mutations are transmitted at rates comparable to those of isolates with high-level-resistance-associated *rpoB* mutations. 

A high frequency of the silent F433F mutation was also observed, interpreted as RIF susceptible in Xpert Ultra, but as RIF-R in LPA, which detected this mutation as inferred. Isolates with synonymous *rpoB* mutations not hybridizing to the WT1, WT3, and WT6 probes highlighted the need to identify mutations with these LPA patterns to avoid false RIF-R classification.

Among INH-R isolates, *inhA* promoter mutations were more prevalent (55.8%) than *katG* mutations (43.8%). The most common mutation was *inhA* C-15T, detected in 52.8% of INH-R cases. Previous studies have also reported a higher prevalence of *inhA* mutations in INH-R than in MDR isolates^7^ ^,^ ^9^ ^,^ ^24^. The frequency of inferred mutations in INH-R isolates (5.6%) was significantly lower than in RIF-R isolates. This finding is consistent with those of other studies^7^ ^,^ ^20^. Furthermore, in our study, the most frequent mutations found in MDR isolates were *rpoB* S450L/*katG* S315T. Both mutations are associated with MDR-TB^3^ ^,^ ^7^ ^,^ ^24^. 

The higher prevalence of the C-15T *inhA* mutation relative to the *katG* S315T mutation is probably explained by the greater number of INH-R than of MDR cases in our study population. A previous investigation revealed a higher prevalence of molecular clustering among isolates carrying *inhA* promoter mutations than among those with *katG* mutations in São Paulo, indicating the sustained transmission of these strains in the community^9^. Hazbón et al.^24^ reported that *katG* S315 mutations increase in frequency as *M. tuberculosis* isolates progress from INH-R to MDR. This pattern may reflect two potential mechanisms: *katG* mutations arise as secondary events following initial INH resistance, or they confer a fitness advantage facilitating survival, transmission, and evolution into MDR strains. Supporting the latter, *inhA* promoter mutations were less frequent in the highly resistant clustered isolates, possibly because of the fitness cost associated with *inhA* overexpression.

We identified three isolates exhibiting no *katG* locus control and WT bands in LPA. Subsequent sequencing revealed that these isolates harbored distinct deletions in the *katG* gene. Although rare, such deletions have been described in clinical settings. A complete deletion of *katG* was previously reported in a clinical isolate from an HIV-infected patient in Italy, conferring high-level resistance to isoniazid (MIC >25.6 µg/mL) without significant impairment in bacterial fitness^25^. Another study has detected small deletions in the 5′ region of *katG*, including a 12-bp deletion in isolates from multiple geographic regions^26^. A study conducted in Brazil reported frequent deletions in *katG* in MDR isolates, supporting the clinical relevance of such structural mutations^27^. Together, these findings suggest that deletions in *katG*, although uncommon, represent a critical mechanism of INH resistance that may evade detection in conventional molecular assays and must be considered in settings with discordant or atypical results.

DST was performed on 39 isolates with inferred mutations, providing a valuable correlation between genotypic and phenotypic resistance. Among these, 21 isolates harbored borderline *rpoB* mutations^15^, all confirmed as RIF susceptible in pDST, suggesting that the critical concentration of 0.5 µg/mL may not be appropriate for detecting low-level resistance caused by these mutations in MGIT, also reported by Rupasinghe et al. (2024)^28^. However, an isolate with the *rpoB* Q432R mutation and another with the *rpoB* P439L mutation were both classified as associated with interim resistance and tested MDR and RIF-susceptible in pDST, respectively. 

Notably, we detected *katG* deletion in three isolates exhibiting no locus and WT bands in LPA, and the isolate tested on pDST was INH-R, confirming the loss-of-function nature of the deletion and its association with resistance^15^. Among the three isolates with inferred mutations in the *inhA* promoter (G-19T, C-20G, and T-8G) tested using pDST, only that with the C-20G mutation, not included in the catalogue, was INH-R. These findings highlight the importance of confirming drug resistance through phenotypic testing.

This study has some limitations. A larger number of isolates need to be analyzed using pDST to ensure a comprehensive elucidation of the variability in genotypic and phenotypic resistance patterns. The estimated prevalence was based solely on laboratory data, and the sociodemographic data of people with drug-resistant TB were not assessed. The results from the Xpert MTB/RIF Ultra assay were evaluated only in cases of unexpected LPA results and when available. Finally, neither the minimum inhibitory concentrations of RIF nor WGS were applied, which could have provided deeper insights into the resistance mechanisms, particularly for isolates with atypical mutations, e.g., large *katG* deletions, and isolates with mutations not included in the WHO catalogue.

Overall, this study highlights the complexity of drug resistance in TB and the critical role of molecular diagnostics, likely requiring a composite of phenotypic methods and other approaches, including sequencing, to guide effective diagnosis and treatment strategies. The detection of novel mutations requires continuous surveillance and adaptation of diagnostic tools to ensure the timely and accurate detection of resistance.
